# Reading From the Crystal Ball: The Laws of Moore and Kurzweil Applied to Mass Spectrometry in Food Analysis

**DOI:** 10.3389/fnut.2020.00009

**Published:** 2020-02-28

**Authors:** Michael Rychlik, Philippe Schmitt-Kopplin

**Affiliations:** ^1^Analytical Food Chemistry, Technical University of Munich, Freising, Germany; ^2^Centre for Nutrition and Food Sciences, Queensland Alliance for Agriculture and Food Innovation, The University of Queensland, Coopers Plains, QLD, Australia; ^3^Research Unit Analytical BioGeoChemistry (BGC), Helmholtz Zentrum München, Neuherberg, Germany

**Keywords:** metabolome databases, analytical chemistry, high resolution, LC-MS sensitivity, structure identification, single molecule detection, dark matter

## Abstract

Predictions about the future knowledge of the “complete” food metabolome may be assayed based on the laws of Moore and Kurzweil, who foresee a technological development on exponential behavior. The application of these laws allows us to extrapolate and predict roughly when each single metabolite in foods could be (1) known, (2) detectable, and (3) identifiable. To avoid huge additional uncertainties, we restrict the range of metabolites to those in unprocessed foods. From current metabolite databases and their coverage over time, the conservative number of all considered food metabolites can be estimated to be 500,000, predicting them being known by around 2025. Assuming these laws and extrapolating the current developments in chromatography and mass spectrometry technology, the year 2032 can be estimated, when single molecule detection will be possible in “routine” mass spectrometry. A possible forecast for the identification of all food metabolites, however, is much more difficult and estimated at the earliest in 2041 as the year when this may be achieved. However, the real prediction uncertainty is extreme and is discussed in the essay presented here.

## Introduction

### Definition of Foodome

In the present “omics” era, one may believe based on literature and instrumental developments that a comprehensive picture of metabolites is known and can be analyzed. However, upon closer inspection and despite immense technological developments, the major fraction still remains unknown. The question arises as to whether a forecast is possible and, if so, when a real comprehensive picture of food metabolites could be available.

Based on the definition of “foodomics” (1), we recently specified “foodome” as being the “collection of all compounds present at a given time in an investigated food sample and/or in a biological system interacting with the investigated food” (2).

As a fraction of the foodome, food metabolome is the set of metabolites in food and is supposed to be accessible using state-of-the-art metabolomics platforms.

The food metabolome has been investigated in a targeted way since the past decades, but the last years have seen an increasing number of efforts to investigate the metabolome by non-targeted methods through applying current high-resolution platforms. Among these, e.g., mass spectrometric detectors have already been compared lately (2).

Besides the continuous efforts to increase the mass exactness and resolving power of a mass spectrometer (MS), developments have been very successful in decreasing the limits of detection (LOD) down to the lower nanogram-per-kilogram level or beyond. The whole analytical pipeline will determine the sensitivity limitation of an analytical approach—from sample conditioning, extraction, and separation toward ionization source to ion manipulations and detections [in the case of liquid chromatography (LC)/MS]. Within all targeted metabolomics platforms, the triple quadrupole (QQQ) mass detectors are now-a-days still ahead in sensitivity compared to time-of-flight (TOF) detectors for the same food sample prepared identically.

Food metabolites are present in food over a wide dynamic range of concentration (from attomolar to molar), and thus their possible detection depends on their individual amount in the considered food, their possible selective isolation out of the matrix in chromatography, their selective ionization potential in the source (and suppression effects with the matrix), and the sensitivity of the mass detector in the mass range observed. Well-known metabolites thus might not be detected by non-targeted food metabolomics although they are present. For instance, the whole group of folate vitamers mostly is missing in a non-targeted study (3), but in the validation of targeted folate methods, we could hardly find any blank natural material that is free from folates (4). Moreover, in our studies on the metabolome of the *Alternaria* fungi, more than 50% of the primary metabolites known in databases are still missing (5).

Only a minority of instrumentally detected molecules are found in databases, not mentioning the isobars and isomers that often are not resolved in the analytical approaches (6).

These findings point to the existence of different categories of “unknown” metabolites or different kinds of metabolic “dark matters” (7).

We see currently a fast development of the analytical equipment (8), and the increase in sensitivity and MS resolution appears to be growing exponentially, which indicates the hypothesis of applicability of the Laws of Moore and Kurzweil in describing the general development of technology over time. In the early 1960's, G.E. Moore hypothesized that the number of transistors in an integrated circuit doubles every 2 years (9), and based thereon, in 1999 Ray Kurzweil proposed the Law of Accelerating Returns.

According to this law, the technological advancement will lead to “…a future period during which the pace of technological change will be so rapid, its impact so deep, that human life will be irreversibly transformed” (10). This period corresponds to the “singularity” or, more precisely, the “technological singularity.”

We hypothesize in this essay that all of these considerations mentioned above lead to the assumption that the laws of Moore and Kurzweil are also applicable to current metabolomics approaches and so to food analysis. Therefore, the application of these laws may allow us to predict roughly when all metabolites in foods will be (1) known, (2) detectable, and (3) identifiable. These predictions are the aims of the present viewpoint. It has to be highlighted that we limit our hypotheses to the development of mass spectrometry with its superior sensitivity and versatility, whereas other spectrometric methods are out of the scope of this study and certainly modulate the outcome in describing the chemical space (11).

## Underlying Data and Categories for Prediction

### Predicting the Magnitude of the Metabolome

If we want to predict the time when the whole food metabolome will be identified, we have to estimate first the approximate number of metabolites that we expect to be identified. Considered herein as “metabolites” are only those direct biological small molecule metabolites of living edible systems, i.e., plant, animal, and microorganism sources—not considered are compounds of chemical abiotic origin or from chemical transformations/recombinations (e.g., hydrolysis, thermolysis, Maillard reaction). For the estimation of these compounds, a look into contemporary metabolite databases is the first step. Several compound databases from different organizations or consortia have been published with different foci in the last decade. A short overview about the number of compounds, focus, and publisher or curator is given in Table 1.

**Table 1:** Examples of important metabolite databases.

**Name/publisher**	**Focus**	**No. of compounds**	**As of**	**Reference/webpage**
PubChem	All human-made chemical compounds	96,110,535	20.08.2019	https://pubchem.ncbi.nlm.nih.gov/
ChemSpider	Chemical compounds from diverse data sources	>71,000,000	12.01.2020	http://www.chemspider.com
Metlin	Endogenous metabolites and xenobiotica	958,000	2017	https://metlin.scripps.edu
Human metabolome database (HMDB)	Human metabolites including conjugates	114,000	2018	http://www.hmdb.ca/
MassBank	High-resolution MS database of metabolites including MS/MS spectra	76,418	12.01.2020	https://massbank.eu/MassBank/Index
KNApSAcK, Nara Institute of Science and Technology	Plant metabolite database	51,179	29.09.2019	http://www.knapsackfamily.com/knapsack_jsp/top.html
LIPID MAPS	Lipids	43,636	10.07.2019	http://www.lipidmaps.org/resources/databases/index.php?tab=lms
Kyoto Encyclopedia of Genes and Genomes (KEGG) database	Metabolites, reactions, enzymes, and genes related to metabolic pathways	18,607	21.08.2019	https://www.genome.jp/kegg/
Golm Metabolome Database (GMD)	GC-EI MS database on plant metabolites	2,222 (metabolites)	17.02.2017	http://gmd.mpimp-golm.mpg.de/dataentities.aspx

The number of compounds included in these databases ranges from a few thousands (GMD) to almost 100 million (PubChem)—the sources ranging from primary metabolites in certain species like humans to all man-made chemical species, respectively. In principle, all of these compounds may be occurring in foods, but the majority of them, being xenobiotica, have very low probability of appearance. For an estimation of the most probable metabolites generally to be expected in foods, it is straightforward to exclude, in a first approximation, all xenobiotica. Moreover, from recent studies on non-enzymatic browning during thermal processing of foods, also termed Maillard reaction, it became obvious that this reaction network results to several tens of thousands of additional metabolites that have started from only a few reactive compounds (11–14). Apart from these, many further reactions of food metabolites like thermolysis, hydrolysis, or the plethora of lipid peroxidation reactions can be assumed to increase the number of metabolites tremendously. In order to keep the numbers manageable, it appears straightforward to just focus on the endogenous compounds of the primary and secondary metabolism of plants, animals, and microorganisms occurring in non-processed foods. This also includes their so-called phase 1 and 2 metabolites. Similarly like not considering metabolites arising from processing such as from Maillard reaction, PubChem, ChemSpider, and Metlin should not be included as they mainly contain xenobiotica. A much better estimation is based on the ~20,000 compounds in KEGG or the currently updated human metabolome database (15) containing 114,000 compounds. For the plant metabolome, more than 200,000 compounds have been estimated (16). Regarding lipids, the most comprehensive database LIPID MAPS lists around 44,000 compounds with *in-silico* predictions running into the hundreds of thousands (17). Some entries of these databases are overlapping, but the extent of overlap is hard to estimate (18). In order to follow a rather worst-case scenario, assuming that there are possibly additional 100,000 still hitherto unknown metabolites, a rough estimation for our most conservative prediction may be 500,000 as the number of all relevant food metabolites. From this rough deduction, the magnitude of uncertainty is obvious and the true number may range from half of this figure to its double, i.e., some hundreds of thousands of additional possible and conceivable metabolites not covered yet.

**Figure 1:**
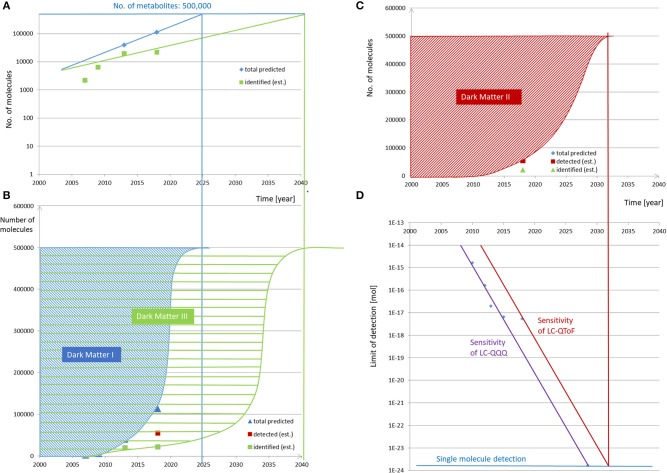
Evolution and prediction of predicted, detected, and identified metabolites over time. **(A,B)** The number of predicted and identified compounds in the HMDB versions 1.0 (2007) and 4.0 (2018) (15) increased in the given time range in a predicted exponential manner and can be further projected accordingly into the future to reach the number of 500,000 metabolites comprising the relevant metabolites in foods, excluding xenobiotica and process-generated compounds. The fractions of not-predicted and not-identified metabolites are termed *dark matter* I and *dark matter* III, respectively. **(C,D)** The evolution of signal sensitivity over time in contemporary LC-QQQ MS instrument in **(D)** is translated into a limit of detection for the given injected amount of molecules, here for the reference compound reserpine. Under the assumption that the limit of detecting reserpine refers to a constant threshold of signal intensity, the limit of detection has been decreasing over time. The development follows an exponential behavior over the last 8 years and can be predicted to follow this path in the future until single molecule detection is reached. As the sensitivity of non-targeted LC-QTOF MS can be expected to be one order of magnitude lower than that of the LC-QQQ, single molecule detection of the former will be reached later. The respective year projected to the current state of detected molecules in HMDB and the estimation of the further evolution to detect the expected number of 500,000 metabolites result in “*dark matter* II,” which is equivalent to the non-detected metabolites over time.

The next step and assumption for our prediction will be the course of time in which these compounds will be discovered. As outlined by Kurzweil (10), many developments, particularly in computing and data handling, follow an exponential evolution. When having a short look at the developments of the databases mentioned above, this time evolution has been reported for HMDB in 2018 and is visualized in Figure 1A. It is evident that, between the years 2007 (HMDB 1.0) and 2018 (HMDB 4.0), the number of compounds increased in this predicted exponential manner (15) and can be further projected accordingly into the future when assuming the Law of Accelerating Returns (10). A further information from HMDB can be concluded, i.e., (i) the number of predicted compounds, (ii) those that have been detected, and (iii) those that have been quantified. This indicates several categories of “unknowns” or “dark matters,” which will be further hypothesized and outlined below.

### Predicting the Performance of Analytical Equipment

In order to detect, identify, and quantify the predicted number of metabolites, the next assumption for our prediction is the forecast on how the performance of the analytical equipment in spectroscopy, spectrometry, and separation sciences, as the analytical pillars, will develop (11). In this respect, we first have to differentiate between targeted and non-targeted metabolomics platforms as outlined above. Second, we have to compile the relevant key performance indicators (KPIs) of the instrumentation such as sensitivity of detection, chromatographic resolution, mass accuracy or MS resolution, and their development over time. For both targeted and non-targeted metabolomics, the primary KPI is the sensitivity of detection, and compiling these data for all analytical approaches would be challenging. As each analytical technology has its own criteria to document sensitivity, for this review, the evolution of sensitivity already published for one analytical platform (2) has been continued.

For instance, development in the sensitivity of mass spectrometric equipment is referenced for the compound reserpine as the signal intensity for a given amount injected into the MS. The ionization efficiency for any other chemical can be different by up to many multitudes and makes it difficult to generalize, but a further assumption to extend our hypothesis will be that the limit of detecting reserpine refers to a constant threshold of signal intensity, which means that the limit of detection is decreasing over time as revealed in Figure 1D.

It is again obvious that the development follows an exponential behavior over the last 8 years and can be predicted to follow this path in the future when assuming the Law of Accelerating Returns set up by Kurzweil. Considering non-targeted LC-QTOF instrumentation, a certain gap in sensitivity is obvious when compared to LC-QQQ, but the equivalent development of increasing sensitivity over time can be assumed. Again it has to be stressed that, within manufacturers' equipments, we will find different performances in sensitivity, but it can be assumed that all of them fall in the same order of magnitude; otherwise, they would not have been competitive in the market up to present.

The next assumption refers to the target sensitivity that the equipment would have to achieve in our prediction. In this respect, a comprehensive detection is required, i.e., a metabolite should be detectable if only one molecule is present in the respective amount of food. This is equivalent to the requirement of single molecule detection. It is clear that the currently detectable few femtograms of reserpine still equals around a few million molecules, but this number can be expected to exponentially decrease according to the trend of the last years (see below) and the assumed Law of Accelerating Returns.

A further KPI to consider would be mass spectrometric resolution, and the exponential increase for TOF instruments has already been reported by Bristow (19).

When comparing the existing lack in sensitivity and resolution of contemporary instruments, the requirement to bridge the sensitivity gap of more than six orders of magnitude toward single molecule detection on the basis of reserpine appears more challenging than the necessity to resolve all metabolites. The resolution currently achieved by the Orbitrap-type of instruments is in the several hundreds of thousands, and 21 Tesla FTICR-MS instruments may already reach up to 50,000,000 and this resolution appears higher than currently needed for food metabolites. Moreover, analytical resolution can also be increased in systems chemical analytics with hyphenations of mass spectrometry with separation sciences or spectroscopy equipments in various dimensionality (i.e., LC-HRMS-NMR or GCxGC-HRMS) (11, 20, 21). Sufficient resolution is already or will soon be achieved within the time range until single molecule detection will be available; single molecule imaging is already reached with techniques such as atomic force microscopy.

### Predicting Analytical Singularity

For this prediction, assuming several hypotheses, the time evolution of the developments in detecting, identifying, and quantifying the metabolome has to be followed up in the future. The target number of metabolites would be hypothetically set at 500,000 in the present study to enable a first evaluation, and when projecting the development of the predicted metabolites in the HMDB, the progress displayed in Figure 1B can be assumed with its exponential projection asymptotically reaching the number of 500,000 in ~2025. This means that, based on such a model, there should no longer be any unknown relevant metabolites in 2025, which appears rather unrealistic when considering the current low percentages of the assigned metabolites in metabolic studies. Until then, these currently unknown compounds may be assigned to the first category of “dark matter,” i.e., “unknowns” or “dark matter I” (Figure 1B).

The next prediction refers to the time when all of these metabolites will be detectable by analytical equipment as resolved features, and this requires the projection of the non-targeted metabolomics to single molecule detection. This is outlined in Figure 1D, which projects the exponential development of the current LOD of 6 × 10^−18^ mol (Table S1) to the LOD being one molecule, i.e., 1.66 × 10^−24^ mol. Assuming this model, single molecule detection on routine MS equipment should be feasible by around 2032. It has to be mentioned that single molecule detection is already available in single particle mass spectrometry (22) or in modern atomic force microscopy (23) with which even the aliphatic or aromatic rings can be visualized. In conclusion, the second category of dark matter would refer to “non-detected” or “dark matter II” and is indicated in Figure 1C. When comparing “dark matter I” with “*dark* matter II,” it has to be kept in mind that the detected molecules may be either unknown or known, i.e., not being present in databases and belonging to “dark matter I” or being present in databases and belonging to not-“dark matter I,” respectively. Therefore, “dark matter I” is not a complete subset of “dark matter II.”

Several weaknesses of this prediction have to be admitted: first, the possibility of single molecule detection does not necessarily imply that all single molecules in a complex food extract will be detectable due to ionization selectivity in mass spectrometry, high dynamic ranges in abundances, presence of enantiomers, etc. As we have to consider ionization suppression for almost all ion sources, the current procedure to circumvent this would be dilution, but this would require an even higher sensitivity. This means that the time point to achieve single molecule detection would be even later, if ever. Apart from general sensitivity considerations, we still have to keep in mind that contemporary MS instrumentation may hardly detect the classes of compounds that are hardly ionizable, and uncovering these will require novel kinds of ion sources. Multiple ionization sources may be useful to cover a broader chemical range and to observe more compound classes. We showed, e.g., in a study with FTICR-MS of complex organic mixtures involving ESI, APPI, and APCI in both positive and negative ionization modes that electrospray ionization in positive mode covers only 30% of the total observable compositional space involving all modalities (6). Moreover, the need to differentiate between all stereoisomers points to the need of novel methods or chromatographic systems for enantioseparation.

### Predicting the Identification of the Whole Food Metabolome

Moreover, one molecule appearing as a resolved analytical signal in the analysis does not necessarily mean that the compound is already identified in structure. In contrast to NMR, MS will only provide the elemental formula and, therefore, allow the molecule to be assigned only tentatively to an unequivocal chemical structure. Moreover, there will still be ambiguity about the identity of the compound even when MS/MS fragmentation, for instance, is applied and gives further evidence about the putative structure. This limitation directly leads to the definition of a third level of uncertainty, i.e., the “non-identified” or “dark matter III.” When comparing “dark matter III” with “dark matter I” and “dark matter II,” it is clear that identified molecules are necessarily known and detected, i.e., members of “dark matter III” are either components of “dark matter I” or “dark matter II” or of both.

For unraveling “dark matter III,” the prediction is even more difficult and more subject to high fluctuations as the direct projection would require data on developments of either all metabolites' extraction, clean-up, and spectroscopic structural assignment by, i.e., NMR or chemical synthesis (if possible in a reasonable time) and commercial availability to that date. However, a rather coarse prediction is possible from the already mentioned HMDB database and here from the number of identified and quantified metabolites developing over the last decade (green curve in Figure 1A). These numbers are much smaller than those of the other categories, and it will take much longer to completely unravel *dark matter* III. If the projection is assumed to be a combination of the current exponential evolution and to run in parallel with the projection of *dark matter* I and II, the time point when all food metabolites will be assignable in food samples could be expected in this model by around 2041 (Figure 1B).

## Discussion

The upmost weakness of the prediction presented here is its ambiguity and requires an intense uncertainty assessment and let us play with numbers. This starts already with the uncertainties referring to the number of metabolites up to the development of the unequivocally identified metabolites. The assumed uncertainty of 50% of the existing number of metabolites would already lead to a time range from 2022 to 2028, respectively, for unraveling *dark matter* I. The speculated uncertain number of 500,000 different metabolites is also relatively conservative, and assuming a number of one million would already lead to 2028 for revealing *dark matter* I and 2060 for *dark matter* II. This is already without considering the plethora of all conceivable metabolites based on chemistry rules that can reach extremely high numbers (11). For instance, a molecule consisting only of carbon, hydrogen, and oxygen with the formula C_n_H_2n_O_n_ at a molecular mass of 500 would have theoretically around 10^16^ calculated possible isomers—and with the formula C_n_H_n_O_n_ an even higher number of 10^20^ isomers, respectively (11). The magnitude of all possible reactions, e.g., during processing or storage (abiotic degradations such as reductions/oxydations, condensation reactions, and polymerizations), is not even counted in this very conservative hypothetical assay. The sensitivity differences between the same type of instrument from different manufacturers may cover one order of magnitude, and the necessity to further dilute the extracts to overcome ionization suppression has an impact on our calculations and thus may expand the time range for uncovering *dark matter* II (i.e., single molecule detection) from 2029 to 2038. For *dark matter* III, the time prediction may range from a projection running in parallel with the best-case scenario for *dark matter* II, i.e., around 2035, to a flat exponential projection of quantified metabolites in HMDB from the database's last update approximating the number of 500,000 not revealed before the end of the twenty-first century. For this development, the law of Kurzweil obviously does not apply within the observed time period. This exemplifies the well-known proverb attributed to Niels Bohr who said that “prediction is very difficult, especially about the future” (24) and thus sets the real limitation of our *wizzard's crystal ball's reading*.

From all of the considerations mentioned above, it seems clear that, in particular, three bottlenecks have to be circumvented before the whole food metabolome can be unraveled: (i) coverage and curation of databases, (ii) sensitivity and resolution of analytical equipment, and (iii) unequivocal metabolite identification.

These issues have to be addressed by the food analytical community, including researchers and developers at academic institutions and instrument manufacturers. A current target for the latter would be the development of ultrasensitive and ultrahigh resolution equipment (in mass spectrometry and multidimensional chromatography). We hope that the law of Kurzweil will be applicable to all of these methodologies.

Further aspects in foodomics involving the quantitation of all of these metabolites have not been mentioned above. This hopefully will be achievable sometime after “all” metabolites have been identified as, with the availability of the needed reference compounds or with the knowledge of unequivocal analytical properties, targeted methods will be available within the next decades.

Another aspect which has been often discussed with respect to analytical development is the miniaturization of the equipment with reduced need in sample amount and the consequent reduction of analysis time. These properties can be expected to also show a similar exponential evolution and would lead to fast, portable, and selective sensors, the development of which is also currently taking place. However, this prediction is out of the scope of this perspective.

At present, all three dark matters exist concurrently and are recognizable for any analytical chemist working in metabolomics, e.g., foodomics. In the further years, the follow-up of the evolution in knowledge predicted for the three dark matters mentioned herein, with the predictions presented here, will indicate the validity of this perspective.

When comparing these predictions with those of the singularity in artificial intelligence (AI) and human intelligence predicted for 2045, it appears likely that by then we will have unraveled at least *dark matter* I and *dark matter* II, and the latter singularity may speed up the unraveling of the remaining *dark matter* III. The help of AI may even have some unpredictable effect on the speed of discoveries and unraveling of all dark matter. Also, after singularity has been reached, the world as we know it now may completely have changed in many other different respects as well.

## Data Availability Statement

All datasets generated for this study are included in the article/Supplementary Material.

## Author Contributions

All authors listed have made a substantial, direct and intellectual contribution to the work, and approved it for publication.

### Conflict of Interest

The authors declare that the research was conducted in the absence of any commercial or financial relationships that could be construed as a potential conflict of interest.
