# Supplementary material for: Reading From the Crystal Ball: The Laws of Moore and Kurzweil Applied to Mass Spectrometry in Food Analysis
Source: Front Nutr. 2020 Feb 28;7:9. doi: 10.3389/fnut.2020.00009 (PMC7058551; doi:10.3389/fnut.2020.00009)
Supplement: Supplementary file 1 [file Table_1.pdf]

Supplementary Material

Table S1

Development of sensitivities of LC-MS/MS equipment from the same manufacturer over the time: signal intensity for 1 pg reserpine and respective limit of detection assuming a minimum signal of 3000 arbitrary units for unequivocal detection in LC-QQQ (Rychlik et al., 2018)

Instrument/type	Year of release	Signal intensity (arbitrary units)	Mol reserpine detected
LCMS 8030/LC-QQQ	2010	3000	$1.65\ 10^{-15}$
LCMS 8040/LC-QQQ	2012	30,000	$1.65\ 10^{-16}$
LCMS 8050/LC-QQQ	2013	250,000	$1.98\ 10^{-17}$
LCMS 8060/LC-QQQ	2015	750,000	$6.6\ 10^{-18}$
LCMS 9030/LC-QToF	2018		$5.467\ 10^{-18}$

LC-QQQ, LC-triple quadrupole MS, LC-QTof, LC-hybrid quadrupole / time of flight
